# Alleviation of Ultrafiltration Membrane Fouling by ClO_2_ Pre-Oxidation: Fouling Mechanism and Interface Characteristics

**DOI:** 10.3390/membranes12010078

**Published:** 2022-01-10

**Authors:** Bin Liu, Meng Wang, Kaihan Yang, Guangchao Li, Zhou Shi

**Affiliations:** 1 Key Laboratory of Building Safety and Energy Efficiency, Ministry of Education, Department of Water Engineering and Science, College of Civil Engineering, Hunan University, Changsha 410082, China; ahxclb@163.com (B.L.); wmnzzd@163.com (M.W.); zhous@hnu.edu.cn (Z.S.); 2 People’s Government Office, Bijiang District, Tongren 554300, China

**Keywords:** ultrafiltration, ClO_2_ pre-oxidation, natural organic matter, separation performance, interfacial free energy, membrane fouling model

## Abstract

In order to alleviate membrane fouling and improve removal efficiency, a series of pretreatment technologies were applied to the ultrafiltration process. In this study, ClO_2_ was used as a pre-oxidation strategy for the ultrafiltration (UF) process. Humic acid (HA), sodium alginate (SA), and bovine serum albumin (BSA) were used as three typical organic model foulants, and the mixture of the three substances was used as a representation of simulated natural water. The dosages of ClO_2_ were 0.5, 1, 2, 4, and 8 mg/L, with 90 min pre-oxidation. The results showed that ClO_2_ pre-oxidation at low doses (1–2 mg/L) could alleviate the membrane flux decline caused by humus, polysaccharides, and simulated natural water, but had a limited alleviating effect on the irreversible resistance of the membrane. The interfacial free energy analysis showed that the interaction force between the membrane and the simulated natural water was also repulsive after the pre-oxidation, indicating that ClO_2_ pre-oxidation was an effective way to alleviate cake layer fouling by reducing the interaction between the foulant and the membrane. In addition, ClO_2_ oxidation activated the hidden functional groups in the raw water, resulting in an increase in the fluorescence value of humic analogs, but had a good removal effect on the fluorescence intensity of BSA. Furthermore, the membrane fouling fitting model showed that ClO_2_, at a low dose (1 mg/L), could change the mechanism of membrane fouling induced by simulated natural water from standard blocking and cake layer blocking to critical blocking. Overall, ClO_2_ pre-oxidation was an efficient pretreatment strategy for UF membrane fouling alleviation, especially for the fouling control of HA and SA at low dosages.

## 1. Introduction

Formed as a result of the interaction between the hydrological cycle, the biosphere, and the lithosphere, natural organic matter (NOM) is commonly found in surface water and groundwater, with diverse distribution patterns [1]. Recently, scholars have observed that the content of NOM in water bodies is increasing, accompanied by severe seasonal changes, and can absorb ultraviolet rays to protect harmful substances, such as pathogens, and reduce the self-purification capacity of surface water [2,3]. In addition, Bond [4] et al. reported that NOM is the precursor of disinfection byproducts such as trihalomethanes (THMs) and haloacetic acids (HAAs). Therefore, NOM can inevitably pose a threat to the operation of water plants. Some components of NOM are collectively referred to as humus, because they have no separate chemical formulae [5]. Lipczynska-Kochany et al. [2] found that humus could be divided into humin, humic acid (HA), and fulvic acid (FA) according to molecular weight, among which HA is considered to be the dominant component of humus. Nevertheless, HA is prone to forming stronger toxic complexes with heavy metals in water [6,7]. Moreover, other types of organics—such as polysaccharides and proteins—also present thorny problems for water treatment. Therefore, it is urgent and necessary to study the removal of NOM in water treatment.

At present, the methods for removing NOM include coagulation [8,9], adsorption [10], advanced oxidation [11], and membrane separation technology [12,13]. However, these methods each have their own shortcomings or application limitations. For example, the use of inorganic coagulants such as aluminum salts or iron salts can only remove part of the NOM. Furthermore, although common adsorbents such as activated carbon can effectively remove NOM, it is difficult to regenerate and recover the adsorption capacity, and the risk of secondary pollution of the water body is also a tricky issue.

Unlike the previous methods, ultrafiltration (UF) can effectively remove low-molecular-weight NOM, and is a small-footprint treatment process, representing an efficient and stable separation technology through size exclusion [14,15]. At present, in order to realize the sustainable development of membranes and improve their anti-pollution properties and durability, many novel materials have been applied, such as sustainable polymer [16], mordenite zeolite [17], poly(vinyl alcohol) [18], etc. However, due to the complex structure, high molecular weight, and strong adsorption of the membrane, the organic molecules cause membrane fouling in the membrane surface and pores more easily. Therefore, during the UF process application, membrane fouling caused by organics should be highlighted [19,20].

To alleviate fouling of ultrafiltration membranes caused by NOM, a variety of pretreatment technologies have been probed in the past several years [21,22,23,24]. Advanced pre-oxidation is considered to be an attractive method among the various pretreatment strategies. For example, the redox point of ozone is E_0_ = 2.076 V, which can effectively change the nature of foulants and degrade membrane foulants. However, due to problems such as strong oxidation and poor selectivity, this pretreatment method may have the potential to deteriorate the quality of ultrafiltered water. The permanganate pre-oxidation method is prone to producing byproducts such as MnO_2_ in the pre-oxidation process, which might reduce the lifespan of the ultrafiltration membrane. As an oxidant with moderate oxidation potential, ClO_2_ can better control its degree of oxidation, and has a better ability to control the generation of disinfection byproducts [25,26].

Zhong [27] et al. reported that the presence of ClO_2_ reduced THMs the most, but not HAAs or HALs, in low-NH3–N wastewater. Shao [28] et al. studied the effects of ClO_2_ pre-oxidation on the formation of dichloroacetonitrile and dichloroacetamide during subsequent chloramination. It was found that ClO_2_ oxidation had inverse effects on DCAN/cAm yields for hydroxybenzamides and tetracyclines. In addition, Gan et al. reported that ClO_2_ selectively reacted with compounds with electron-rich moieties, such as phenols, anilines, and thiols in the case of organic compounds [29]. Therefore, the discussion of the potential of ClO_2_ as UF pretreatment for NOM in water has practical and scientific significance. This paper investigated the possibility of applying ClO_2_ for pre-oxidation of the UF process.

In this study, the use of ClO_2_ as a pre-oxidation method was employed to explore the mitigation effect and mechanism of membrane fouling, as well as the water purification efficiency. Three typical organic compounds—humic acid, sodium alginate, and bovine serum protein—were used as model foulants to simulate various components in surface water. Initially, membrane fouling and rejection of organic compounds were investigated. In addition, the interface characteristics of the fouled membranes—including particle size distribution, zeta potential, interfacial free energy, and morphology—were comprehensively investigated.

## 2. Materials and Methods

### 2.1. Preparation of Experimental Sewage

Humic acid (HA), sodium alginate (SA; representative of polysaccharides), and bovine serum protein (BSA; representative of proteins), which obtained from alading company (Shanghai, China), were selected for use in the raw sewage for the membrane filtration experiments. In order to obtain 1 g/L of humic acid stock solution, 1 g of solid humic acid powder was accurately weighed on a high-precision electronic balance and then dissolved in a 1000 mL beaker together with 800 mL of sodium hydroxide solution with a concentration of 0.01 mM/L. After that, the pH was adjusted to 7.0, and the whole process was carried out again on a magnetic stirrer. In order to obtain 1 g/L of sodium alginate and bovine serum protein, 1 g of sodium alginate and bovine serum protein solid powder were each weighed in a beaker, and then placed in a magnetic stirrer with heating and stirring for 24 h; lastly, the volume was adjusted to 1000 mL. The above model foulant stock solution was stored in a refrigerator at 4 °C. For the oxidation and filtration tests, the HA, SA, and BSA were all diluted to 5 mg/L, and the concentration of the three organic compounds in the mixed solution was also 5 mg/L.

### 2.2. Experimental Setup

The dosages of AR-grade ClO_2_ were 0.5, 1, 2, 4, and 8 mg/L. The experimental sewages were pre-oxidized for 90 min. Then, 60 mL of solution was taken out for filtration experiments. Flat ultrafiltration membranes of polyethersulfone (PES, 100 kDa) were used in the filtration experiments, which were obtained from the MICRODYN-NADIR company (Wiesbaden, Germany). The ultrafiltration device maintained a constant pressure (100 kPa) with a dead-end ultrafiltration system, without stirring of the feed solution. The device consists of an ultrafilter cup, an electronic balance (NV2201ZH, Auhaus, Shanghai, China), a nitrogen cylinder, a pressure gauge, a control valve, and a computer terminal. The cleaned ultrafiltration membrane was placed at the bottom of the ultrafiltration cup, and the pressure in the ultrafiltration cup was kept low enough that the ultrafiltration cup did not leak air or water. Under this pressure, the liquid in the ultrafilter cup flowed through the silicone tube into the beaker placed on the electronic balance. The balance was set to record the weight data at intervals of 3 s. The weight data recorded by the electronic balance were then transmitted to the computer terminal through the data line in order to obtain the ultrafiltration weight data.

### 2.3. Membrane Fouling Assessment and Mechanism Analysis

The resistance of the ultrafiltration membrane is composed of inherent resistance, reversible resistance, and irreversible resistance [30]. It is generally believed that the reversible resistance is caused by the cake layer on the membrane surface. The reversible resistance can be greatly reduced by gently wiping the cake layer on the membrane surface with wet sponge. Irreversible resistance is usually caused by membrane pore narrowing. The specific calculation can be deduced by the Darcy formula [31,32], as shown in Equation (1):

(1) $$J=\frac{∆P}{\mu (R_{i}{+R}_{b}{+R}_{c})}=\frac{∆P}{{\mu R}_{\mathrm{tot}}}$$

where J represents the filtration flux (L/m^2^·h), μ represents the dynamic viscosity (Paxs), ΔP represents the operating pressure (Pa), R_tot_ represents the total membrane resistance (m^−1^), R_i_ represents the membrane’s inherent resistance (m^−1^), R_b_ represents irreversible resistance (m^−1^), and R_c_ represents the reversible resistance (m^−1^). Ultrapure water filtration was used before each filtration until the flux was stable, and the steady membrane flux was denoted as J_0_. All filtration tests were performed at least 3 times. The R_i_ can be calculated according to Equation (2):

(2) $$R_{i}=\frac{∆P}{{\mu J}_{0}}$$

At the end of filtration, the average flux of the last 3 mL was denoted as J_1_ to calculate the total membrane resistance, as shown in Equation (3):

(3) $$R_{\mathrm{tot}}=\frac{∆P}{{\mu J}_{1}}$$

In order to calculate the irreversible resistance of the membrane, after each filtration cycle, the ultrafiltration membrane was reset in the ultrafiltration device after gently wiping the cake layer on the membrane surface with a wet sponge under water flushing, and pure water was filtered again, i.e., the stable membrane flux after cleaning was obtained, denoted as J_2_. Therefore, the irreversible resistance R_b_ can be calculated according to Equation (4). The reversible resistance can be calculated by subtracting the inherent resistance and irreversible resistance from the total membrane resistance.

(4) $$R_{b}=\frac{∆P}{{\mu J}_{2}}-\frac{∆P}{{\mu J}_{0}}\;$$

In order to further investigate the membrane fouling mechanism during ultrafiltration, four classical fouling models were introduced, including a complete blocking model, critical blocking model, standard blocking model, and cake layer filtration model. The complete blocking model assumes that the pores of the ultrafiltration membrane are completely blocked, so that the transmembrane resistance increases. The standard blocking model assumes that the small-particle-size foulants adhere to the inner sides of the membrane pores, narrowing them and reducing the flow capacity. The critical blocking model is between the complete blocking model and the standard blocking model. The cake layer filtration model refers to the formation of a cake layer by foulants deposited on the membrane surface. Ho and Zydney [33]’s differential form model was introduced in this study to analyze and fit the flux data. The advantage of this method is that it can more intuitively analyze the fouling mechanism under different filtration times or volumes. MATLAB was used to conduct mathematical modeling for the imported data, and cubic polynomial fitting was performed for the flux data. The formulae were as follows:

(5) $$\frac{d^{2}t}{{\mathrm{dV}}^{2}}=k(\frac{\mathrm{dt}}{\mathrm{dV}}{)}^{n}=-\frac{1}{J^{3}A^{2}}\frac{\mathrm{dJ}}{\mathrm{dt}}$$

(6) $$\;\;\frac{\mathrm{dt}}{\mathrm{dV}}=\frac{1}{\mathrm{JA}}$$

(7) $$n=\frac{d\left[\log\left(\frac{d^{2}t}{{\mathrm{dV}}^{2}}\right)\right]}{d\left[\log\left(\frac{\mathrm{dt}}{\mathrm{dV}}\right)\right]}$$

where t represents the filtration time (s), J represents the filtration flux, and V represents the total filtration volume (mL). After finding the turning point by fitting the curve, the data before and after the change point were fitted respectively to determine the leading mechanism in different groups of fouling, according to the obtained N value: when n = 2, it is mainly a complete blocking model; when n = 1.5, standard blocking plays the dominant role; when n = 1, it is critical blocking; when n = 0, it is cake layer filtration [34].

### 2.4. Analytical Methods

The ultraviolet absorbance (UV_254_) was determined using an ultraviolet spectrophotometer (T6, PUXI, Beijing, China), and dissolved organic carbon (DOC) was determined using a total organic carbon analyzer (TOC-VCSH, Shimadzu, Kyoto, Japan). pH was measured with a pH meter (868-2, Orion, Shanghai, China). Because the humic and tryptophan compounds in the target foulant have fluorescent excitation properties, the fluorescent compounds in the solution can be determined using the three-dimensional fluorescence excitation–emission matrix (EEM) spectra. A fluorescence spectrometer (F-7000, Hitachi, Tokyo, Japan) was used for determination. The excitation light (Ex) wavelength range was 200–450 nm, and the emission light (Em) wavelength range was 250–550 nm; the scanning intervals were 5 nm and 1 nm, respectively. The sample solution was filtered through a 0.45 μm microfiltration membrane, and the pH value was adjusted to 7.0 before the EEM test.

The contact angle of the liquid drops to the membrane surface was measured using a contact angle tester (JYSP-360, Beijing, China). Based on the extended Derjaguin–Landau–Verwey–Overbeek (XDLVO) theory, diiodomethane, ultrapure water, and glycerol were selected as test liquids to calculate the interfacial free energy. Computational methods of cohesion and adhesion free energy can be found in a previous work [35]. Scanning electron microscopy (Gemini SEM 300, Zeiss, Germany) was used to observe the morphology and microstructure of foulant particles on the ultrafiltration membrane surface, and the detailed working conditions were similar to those in previous studies [36,37].

## 3. Results

### 3.1. Effect of ClO_2_ Pre-Oxidation on UF Membrane Fouling Alleviation

#### 3.1.1. ClO_2_ Pre-Oxidation for HA Fouling

**Figure 1:**
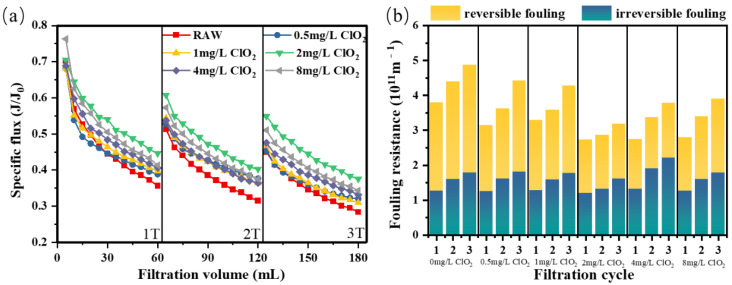
(**a**) Flux curve and (**b**) fouling resistance of the ultrafiltration membrane under different pre-oxidation dosages for HA.

The alleviating effect of ClO_2_ pre-oxidation on ultrafiltration membrane fouling is shown in Figure 1. As shown in the figure, the decrease in membrane flux caused by raw humic acid water was more obvious than that after ClO_2_ pre-oxidation. At the end of the first, second, and third filtration cycles, the specific flux (J/J_0_) decreased to 0.36, 0.31, and 0.28, respectively. ClO_2_ pre-oxidation significantly improved the flux, and the optimal flux mitigation was obtained when the dosage was 2 mg/L. At the end of each cycle of 2 mg/L ClO_2_, the specific flux J/J_0_ rose to 0.45, 0.40, and 0.38, respectively. However, when the ClO_2_ dosage was increased to 8 mg/L, the J/J_0_ at the end of each filtration cycle was decreased to 0.41, 0.37, and 0.34, respectively. Compared with the dosage of 2 mg/L, the ability to recover J/J_0_ at 8 mg/L was limited; thus, it could be seen that there was no positive correlation between ClO_2_ dosage and flux recovery, which might be related to the membrane fouling mechanism [38].

In addition, after primary and secondary backwashing, the flow recovery rate was lower by 73% and 51%, respectively, indicating that the flow recovery rate of hydraulic backwashing was effectual, and that reversible fouling was dominant in HA fouling. As shown in Figure 1b, the total fouling resistance and reversible fouling resistance caused by raw humic acid water were the highest in the three cycles, and the total fouling resistance of different ClO_2_ dosage groups decreased at first, and then increased over three filtration cycles, during which the optimal fouling resistance alleviation was achieved when the dosage was 2 mg/L. On the other hand, the proportion of irreversible fouling resistance increased, and the decline in the degree of resistance was relatively lower—even when the dosage was 4 mg/L, there was an increase at the end of the third filtration cycle. These results indicated that ClO_2_ pre-oxidation had a good effect on improving permeate flux and alleviating reversible fouling during humic acid filtration, and that the filtration performance might be reversely deteriorated when dosed with excessive ClO_2_. As experiments by Gan et al. showed, the yield of ClO_2_ in humus depends on the dose and pH value of ClO_2_ [39].

#### 3.1.2. ClO_2_ Pre-Oxidation for SA Fouling

**Figure 2:**
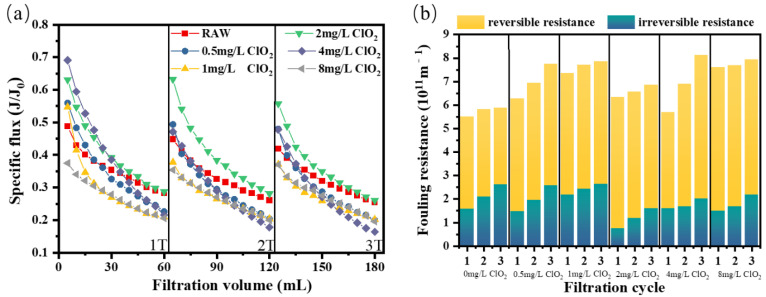
(**a**) Flux curve and (**b**) fouling resistance of the ultrafiltration membrane under different pre-oxidation dosages for SA.

As depicted in Figure 2a, the decrease in membrane flux caused by SA is more obvious than that caused by humic acid. The specific flux remained flat or even lower at the end of each cycle as the ClO_2_ dose increased from 0 mg/L to 8 mg/L. When the ClO_2_ dosage was 2 mg/L, although the flux decrease was relieved to some extent, the specific flux at the end of each filtration cycle was close to the value of sodium alginate raw water. Compared with humic acid, flux recovery of sodium alginate after hydraulic backwash was more apparent. As shown in Figure 2b, sodium alginate mainly caused severe reversible resistance, with a higher proportion than that in humic acid, which could be eliminated by high-intensity backwashing. With the dosage of ClO_2_ above 2 mg/L, the irreversible fouling resistance decreased, indicating that ClO_2_ pretreatment alleviated the irreversible fouling caused by sodium alginate to a certain extent. On the other hand, the reversible fouling caused by sodium alginate increased significantly with the dosing of ClO_2_, which was the most serious when the dosage was 8 mg/L, showing an increased reversible fouling resistance of 2.19 × 10^11^ m^−1^ compared with the sodium alginate raw water.

It could be seen that reversible fouling with pretreatment of ClO_2_ played a leading role in membrane fouling induced by sodium alginate. This was consistent with previous research results, which could be attributed to the fact that sodium alginate can rapidly form a gel layer or cake layer on the membrane surface, causing membrane surface fouling [40].

#### 3.1.3. ClO_2_ Pre-Oxidation for BSA Fouling

**Figure 3:**
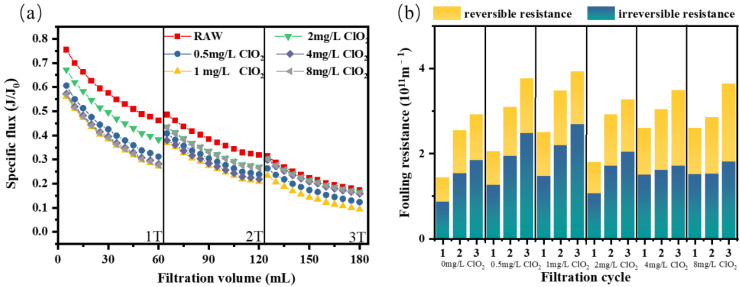
(**a**) Flux curve and (**b**) fouling resistance of the ultrafiltration membrane under different pre-oxidation dosages for BSA.

The specific flux of different ClO_2_ doses dropped sharply during the initial filtration cycle, after which the flux curve became smooth in the third cycle (Figure 3a). At the end of the first, second, and third filtration cycles, bovine serum protein caused a severe decrease in flux, with specific fluxes dropping to 0.46, 0.32, and 0.17, respectively. Moreover, the flux was difficult to recover after physical cleaning. Although the flux in each cycle increased slightly after hydraulic flushing with the dosing of ClO_2_, the flux decreased more significantly with the increase in the dose during the first two filtration cycles, indicating that ClO_2_ pre-oxidation had no alleviating effect on flux reduction caused by bovine serum protein.

As shown in Figure 3b, irreversible and reversible fouling resistance caused by BSA at the end of filtration accounted for 62.8% and 27.2% of the total resistance, respectively. It could be concluded that irreversible resistance played the dominant role in membrane fouling caused by bovine serum protein. Thus, the membrane flux was not well alleviated after hydraulic cleaning; however, after the pre-oxidation with ClO_2_ (0.50–8 mg/L), both reversible and irreversible fouling were not only not alleviated, but even aggravated, at dosages below 1 mg/L. In addition, Cheng et al.’s experimental results also showed that lower doses of PMS as an oxidant aggravated membrane fouling by BSA [38]. These results indicate that ClO_2_ pre-oxidation cannot improve the flux and reduce the fouling resistance during bovine serum albumin filtration. A possible reason for the limited fouling mitigation effect of pre-oxidation on BSA—especially compared to the mitigation effect for HA and SA—is the high molecular weight of BSA, which cannot be efficient degraded by a low-dosage oxidation process.

#### 3.1.4. ClO_2_ Pre-Oxidation for NOM Fouling

**Figure 4:**
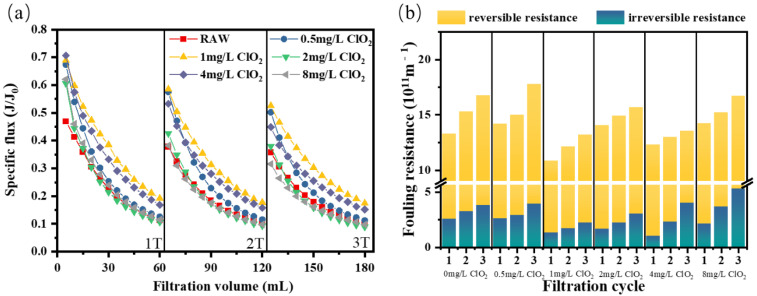
(**a**) Flux curve and (**b**) fouling resistance of the ultrafiltration membrane under different pre-oxidation dosages for NOM.

In actual water treatment, humus, proteins, and polysaccharides generally exist together. Therefore, in order to further explore the influence of ClO_2_ as a pre-oxidant on membrane fouling caused by compound foulants, raw water mixed with humic acid, sodium alginate, and bovine serum protein was prepared. Figure 4 shows the influence of ClO_2_ pre-oxidation on the membrane fouling of simulated natural water. At the end of three cycles of direct filtration, the specific flux of ultrafiltration membrane decreased to 0.11, 0.10, and 0.09, respectively. As can be seen from the figure, when the dose was 1 mg/L, ClO_2_ pre-oxidation had the most obvious effect on improving flux compared with other doses, which was more prominent in the second and third filtration cycles.

Unlike the treatment of model foulants, the membrane flux could be effectively recovered by hydraulic flushing. As shown in Figure 4b, similar to the treatment of raw water with individual forms of organic matter, the mitigation of membrane fouling was not improved with the increase in oxidizer dosage. On the contrary, low doses (1–4 mg/L) could effectively alleviate both reversible and irreversible resistance. These results showed that low ClO_2_ pre-oxidation could not only improve the specific flux of mixed simulated natural water, but also reduce the membrane fouling—especially the irreversible fouling [41].

### 3.2. Effect of ClO_2_ Pre-Oxidation on Purification Performance

**Figure 5:**
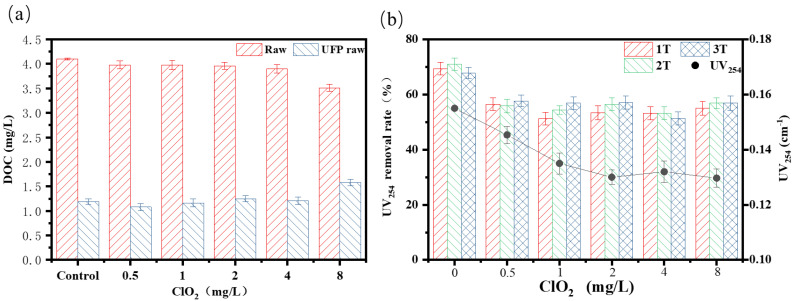
UV_254_ detection values and DOC concentrations at different dosages of pre-oxidation: (**a**) DOC; (**b**) UV_254_.

Figure 5a shows the influence of pre-oxidation of ClO_2_ at different dosages on DOC in raw water. As shown in the figure, with the increase in ClO_2_ dosage (0.5–8 mg/L), the influent DOC values were 3.98 mg/L, 3.97 mg/L, 3.96 mg/L, 3.90 mg/L, and 3.51 mg/L, respectively. It was found that the dosing of ClO_2_ caused a very limited improvement in DOC rejection, which is consistent with the results of previous studies on ozone degradation of organic foulants [41]. Compared with DOC removal, the UV_254_ value changed more obviously, which was because unsaturated bonds and aromatic rings preferentially reacted with oxidants and free radicals in water (Figure 5b) [42]. As shown in the figure, with the increase of ClO_2_ dosage, the UV_254_ value of raw water decreased from 0.155 to 0.129, and the removal rate was 16.70%. When the dosage range of ClO_2_ was between 2 and 8 mg/L, the absorbance remained virtually unchanged. In general, after adding ClO_2_, the removal rate of UV_254_ by the ultrafiltration membrane slightly decreased. When compared with previous work, it can be confirmed that ClO_2_ oxidation has a better removal effect than chlorine oxidation in terms of DOC and UV_254_ [43].

**Figure 6:**
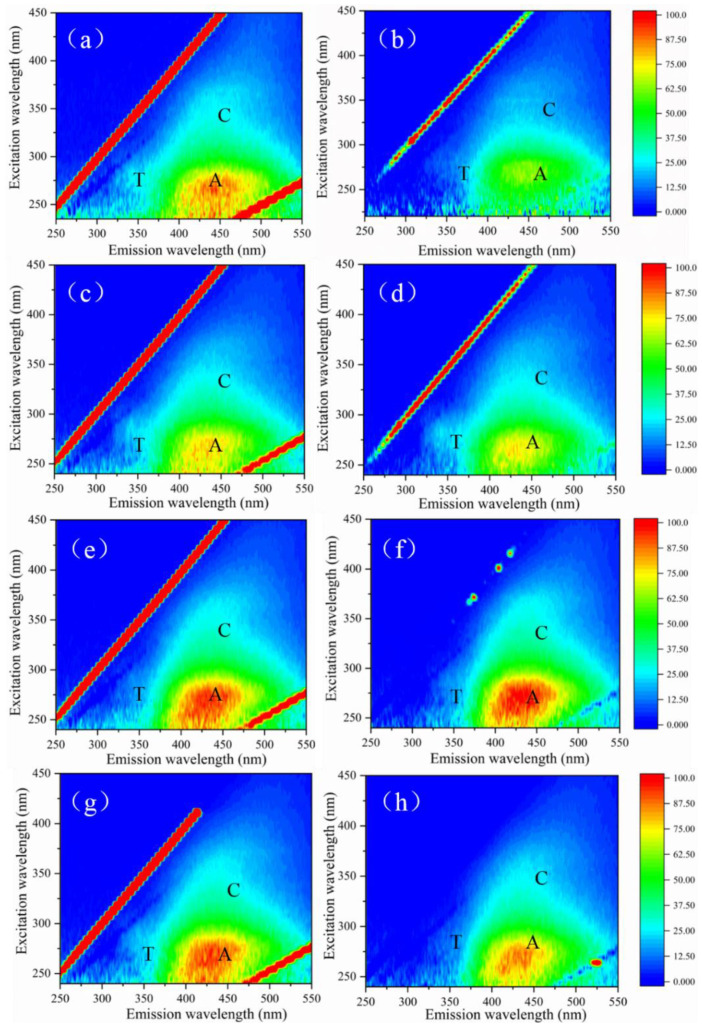
EEM spectra of samples under different pre-oxidation conditions: (**a**) humic acid raw water; (**b**) effluent of humic acid; (**c**) 2 mg/L ClO_2_ pre-oxidation; (**d**) effluent of 2 mg/L ClO_2_ pre-oxidation; (**e**) 4 mg/L ClO_2_ pre-oxidation; (**f**) effluent of 4 mg/L ClO_2_ pre-oxidation; (**g**) 8 mg/L ClO_2_ pre-oxidation; (**h**) effluent of 8 mg/L ClO_2_ pre-oxidation.

The humic and tryptophan compounds in the target contaminant have fluorescent excitation properties. Therefore, these two substances in the solution can be determined using the EEM. Three-dimensional fluorescence spectra of effluents under different treatment conditions are shown in Figure 6. The three-dimensional fluorescence spectrograms have three distinct characteristic peaks: T, A, and C. The characteristic peaks of humus-like organics are generally considered as A and C, which mainly appear in the range of excitation wavelength 250–350 nm and emission wavelength 350–500 nm, while the characteristic peaks of tryptophan protein (T) mainly appear in the range of excitation wavelength 225–280 nm and emission wavelength 310–340 nm [44]. As shown in Figure 6a, humic acid raw water had an obvious characteristic peak A at Ex/Em = 250–400/380–540 nm, representing humus-like organics. When the dosage of ClO_2_ was 2 mg/L, the fluorescence value of humus-like organics decreased. With the increase of ClO_2_ dosage (4–8 mg/L), the fluorescence response value of peak A increased appropriately, and reached a peak value at the dosage of 4 mg/L.

This study has shown that some functional groups that can stimulate fluorescence are hidden in humus water samples, and these hidden functional groups can be activated under the action of oxidants, resulting in the increase in the fluorescence values of humic analogs [45]. As can be seen from Figure 6b,d,f,h, peak A intensity weakened after filtration by the PES ultrafiltration membrane, indicating that the ultrafiltration membrane has certain interception and adsorption capacity for small-molecule humus-like organics that can stimulate fluorescence [46].

**Figure 7:**
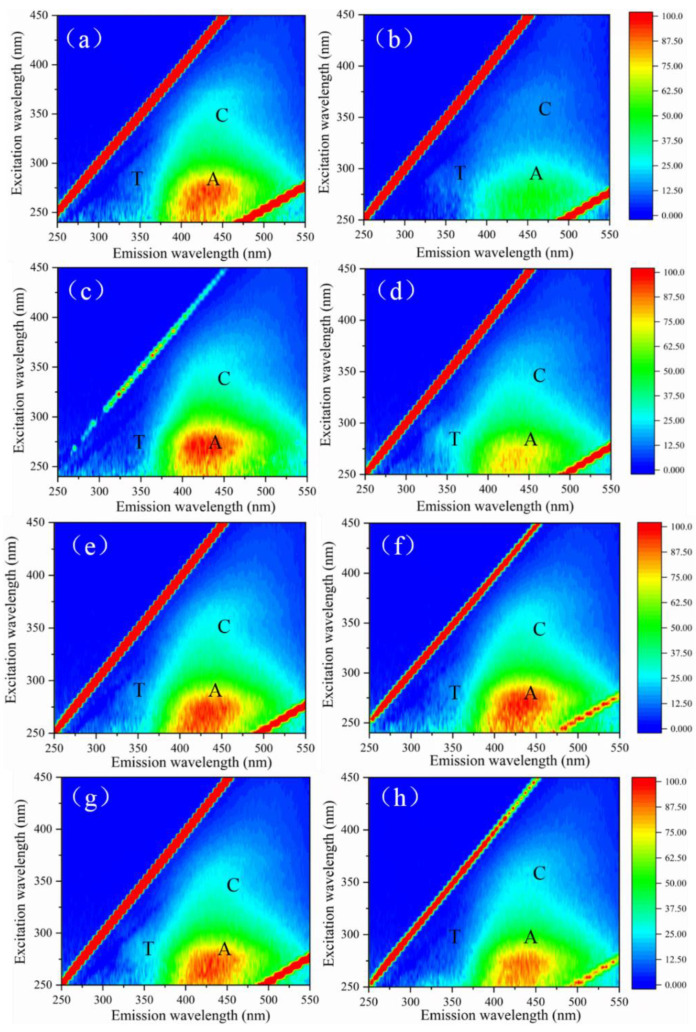
EEM spectra of samples under different pre-oxidation conditions: (**a**) simulated natural water; (**b**) effluent of simulated natural water; (**c**) 1 mg/L ClO_2_ pre-oxidation; (**d**) effluent of 1 mg/L ClO_2_ pre-oxidation; (**e**) 4 mg/L ClO_2_ pre-oxidation; (**f**) effluent of 4 mg/L ClO_2_ pre-oxidation; (**g**) 8 mg/L ClO_2_ pre-oxidation; (**h**) effluent of 8 mg/L ClO_2_ pre-oxidation.

Figure 7 shows the effects of the ClO_2_ ultrafiltration process on fluorescent substances of simulated natural water. As with the results of humic acid, the addition of ClO_2_ reduced the intensity of peak A and produced an excitation effect on the fluorescent substances concealed in raw water. When the ClO_2_ dose was 8 mg/L, peak A, representing humic substances, was significantly reduced, and the fluorescence value of peak A after UF was also lower than the other conditions, corresponding to the result of UV_254_, indicating that the ClO_2_ preferentially attacked unsaturated bonds and aromatic ring substances in humic acids [42].

### 3.3. Effect of ClO_2_ Pre-Oxidation on Interface Characteristics and Fouling Mechanisms

**Figure 8:**
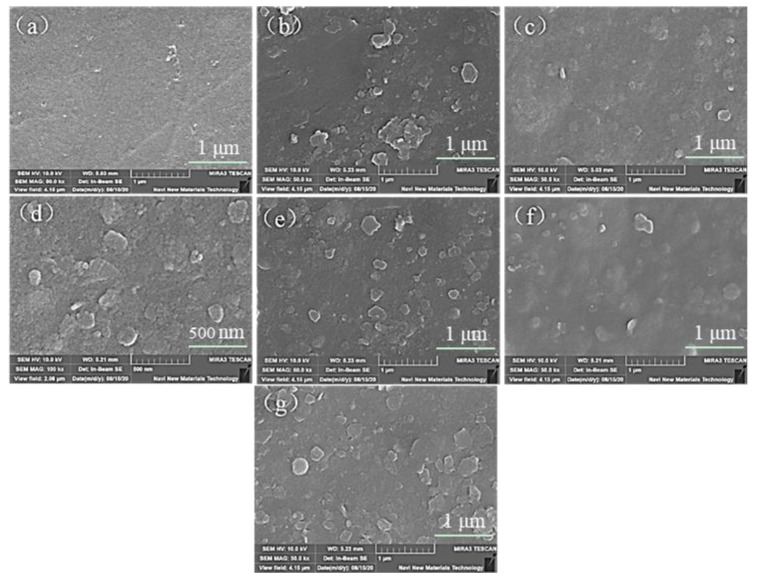
SEM images of pollutant morphology on membrane surfaces under different ClO_2_ dosages: (**a**) pristine membrane; (**b**) membrane having filtered humic acid; (**c**) membrane having filtered humic acid after 2 mg/L ClO_2_ pre-oxidation; (**d**) membrane having filtered humic acid after 8 mg/L ClO_2_ pre-oxidation; (**e**) membrane having filtered simulated natural water; (**f**) membrane having filtered simulated natural water after 2 mg/L ClO_2_ pre-oxidation; (**g**) membrane having filtered simulated natural water after 8 mg/L ClO_2_ pre-oxidation.

The surface of the ultrafiltration membrane was characterized by scanning electron microscope (SEM), and the mechanism of ultrafiltration membrane fouling caused by ClO_2_ was further analyzed by fitting the interfacial free energy and membrane fouling model. As shown in Figure 8a, the surface of the original ultrafiltration membrane was clean and smooth, with no accumulation of foulant. However, after filtration of humic acid and simulated natural water, the surface morphology of the membrane was obviously different due to the accumulation of foulants [38]. The membrane surfaces after filtration of HA and simulated natural water are shown in Figure 8b,e, respectively, where we can observe obvious particle deposition on the membrane surfaces and a relatively dense fouling layer/cake layer. Figure 8c,d show the SEM images of membrane surfaces having filtered pre-oxidized humic acid solution with ClO_2_ dosages of 2 mg/L and 8 mg/L, respectively. It can be seen that compared with Figure 8b, the membrane surface of the two pretreatment conditions was relatively smooth, and the surface foulant particles were also reduced in quantity.

As shown in Figure 8f, when the dosage of ClO_2_ was 2 mg/L, the foulants on the membrane surface had a boundary outline except for a few places, and the rest of the membrane surface was relatively smooth. When the ClO_2_ dosage was 8 mg/L, the membrane surface showed a large accumulation of foulants; this may have been due to the agglomeration of organic substances in raw water after pre-oxidation, which reduced the membrane water capacity and increased the membrane resistance, which was not conducive to the stable operation of the membrane.

The XDLVO theory was used for interface analysis, and the condensation free energy and adhesion free energy of the ultrafiltration membrane surface with various ClO_2_ doses were calculated. The results are shown in Table 1 and Table 2, in which ∆G^LW^, ∆G^AB^, and ∆G^EL^ are the van der Waals force condensation/adhesion free energy, polar force condensation/adhesion free energy, and electrostatic condensation/adhesion free energy, respectively. In addition, ∆G_131_ represents the condensation free energy between the interfaces, ∆G_132_ represents the adhesion free energy of the interface, and their values are calculated through the various interface forces. “∆G > 0” indicates that the material interface is in a stable state, which is repulsive; “∆G < 0” indicates that the material interface is in an unstable state, and is attractive [47]. As shown in Table 1, ∆G^LW^ and ∆G^EL^ slightly changed with the increase in ClO_2_ dosage, indicating that polar force dominated. In addition, the ∆G_131_ was −5.36 mJ/m^2^, ∆G^AB^ was −1.63 mJ/m^2^, and the value of ∆G^AB^ increased to 14.10 mJ/m^2^ with the increase in the ClO_2_ dose (from 2 mg/L to 8 mg/L), indicating that the surface polarity increased and the hydrophilicity was improved. However, when ClO_2_ was added, the adhesion free energy of ∆G_132_ between humic acid and the UF membrane increased from −8.28 to 8.91, indicating that the attraction between humic acid and the UF membrane changed to repulsion.

As shown in Table 2, the ∆G^EL^ and ∆G^LW^ between the ultrafiltration membrane and the simulated natural water did not change much with the dosage of ClO_2_, which was still dominated by polar force. When the ClO_2_ dosage was 8 mg/L, unlike with humic acid, the value of ∆G_131_ was −13.16 mJ/m^2^, indicating that the foulants were in a state of mutual attraction. At the same time, the value of ∆G_132_ was −0.07 mJ/m^2^, indicating that the adhesion free energy between the foulants and the membrane was small and attractive, facilitating deposition on the membrane surface. This confirmed that the obvious foulant profile of the simulated natural water on the membrane surface in the SEM figure was the result of mutual attraction between the mechanical mixture and the ultrafiltration membrane, and was consistent with the obvious attenuation of membrane flux and serious membrane fouling.

**Figure 9:**
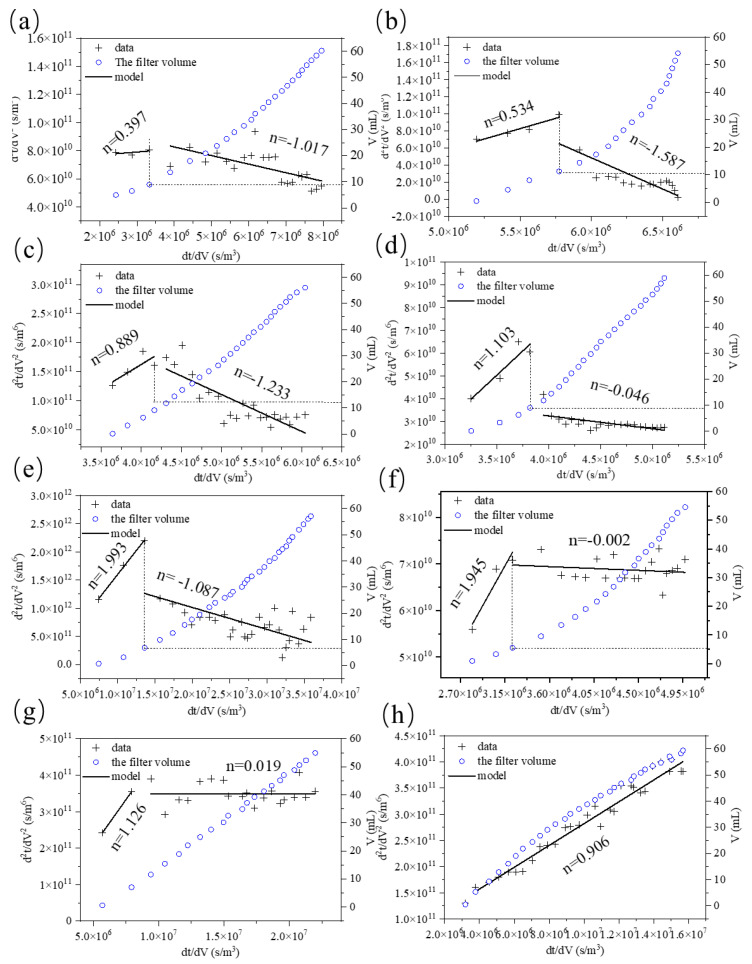
Data and fitting curves (membrane d^2^t/dV^2^ and dt/dV) for ultrafiltration of organic foulants with different ClO_2_ dosages: (**a**) HA without pre-oxidation; (**b**) HA with 2 mg/L ClO_2_ pre-oxidation; (**c**) SA without pre-oxidation; (**d**) SA with 2 mg/L ClO_2_ pre-oxidation; (**e**) BSA without pre-oxidation; (**f**) BSA with 2 mg/L ClO_2_ pre-oxidation; (**g**) simulated natural water without pre-oxidation; (**h**) simulated natural water with 2 mg/L ClO_2_ pre-oxidation.

In order to further investigate the effect of ClO_2_ pretreatment on the membrane fouling mechanism, the membrane fouling model based on d^2^t/dV^2^ and dT/dV (Ho and ZyD-Ney, 2000) was adopted, where the t and V represent the membrane filtration time and the total membrane filtration volume, respectively. As can be seen from Figure 9, the values of “D^2^”, “T/D”, “V^2^”, and “DT/dV” decreased significantly after ClO_2_ pre-oxidation. However, the value of “DT/dV” increased during the treatment of BSA, indicating that the filtration time increased during ultrafiltration. It was confirmed that severe membrane fouling was produced during the pretreatment of BSA with ClO_2_, which is consistent with its flux curve.

As shown in Figure 9a, the n value of HA without pre-oxidation was 0.40 at the initial stage of filtration, and −1.02 at the later stage of filtration. At this time, the fouling mechanism of the membrane surface caused by the blocking of membrane holes changed [48], which is consistent with Wang’s results of UV/chlorine treatment of humic acid [43]. It could be concluded that the membrane fouling mechanism was mainly a result of membrane hole blockage and the cake layer. Figure 9b shows the fitting curve of HA after pre-oxidation with 2 mg/L ClO_2_; it can be seen that the fitting curve is similar to that in Figure 9a, indicating that the fouling mechanism was consistent with and without ClO_2_ pre-oxidation. When SA was pre-oxidized at 2 mg/L ClO_2_, the membrane fouling mechanism was not changed, although the fouling was alleviated. It is noteworthy that after oxidizing simulated natural water with 2 mg/L ClO_2_, the fouling mechanism changed from cake layer fouling to critical blocking, which alleviated membrane fouling.

## 4. Conclusions

In this work, the effects of ClO_2_ pre-oxidation on the treatment of various typical organic foulants—and their mixture simulating natural water—with an ultrafiltration membrane were investigated, and the fouling mechanism was analyzed by membrane surface morphology, interfacial free energy, and membrane fouling models. It was found that this method was not only low-cost and easy to operate, but also could effectively alleviate membrane fouling caused by simulated natural water in the appropriate dosage range. The main conclusions are as follows:

ClO_2_ pre-oxidation at a lower concentration (1–2 mg/L) can alleviate the decrease in membrane flux caused by humus, polysaccharides, and mixed organic foulants, among which the highest traffic recovery rate reached 73%, 89%, and 86%, respectively, although its effect in terms of reducing membrane irreversible resistance was limited. In addition, the experimental results showed that ClO_2_ pre-oxidation faced some difficulty in alleviating the membrane flux decrease caused by protein substances. Furthermore, the removal effect of DOC in raw humic acid water by ClO_2_ dosing was not ideal. ClO_2_ oxidation activated the hidden functional groups in the raw water, resulting in an increase in the fluorescence value of humic analogs and a good removal effect on the fluorescence intensity of BSA. In general, the dosage of ClO_2_ was inversely proportional to the mitigation effect of membrane fouling, as well as the irreversible fouling, in the treatment of simulated natural water.

The interfacial free energy analysis showed that the attraction between organics increased, and they easily deposited onto the membrane surface, resulting in a serious flux decline and membrane resistance increase when the dosage was 8 mg/L. However, at 1 mg/L pre-oxidation, the polarity force between the membrane and organic matter could be improved, thus increasing the repulsion force and, in turn, alleviating membrane fouling.

The fitting results of the membrane fouling model showed that ClO_2_ could not effectively change the fouling mechanism caused by typical organic matter in water, and that the pre-oxidation of BSA could accelerate the formation of the cake layer, thus prolonging the membrane filtration duration. However, at a low concentration (1 mg/L), the membrane fouling mechanism induced by simulated natural water could change from standard blocking and cake layer blocking to critical blocking.

## Figures and Tables

**Table 1:** Free energy of interfacial condensation and adhesion of the ultrafiltration membrane surface, caused by humic acid (mJ/m^2^).

Dosage (mg/L)	$∆G_{131}^{\mathrm{LW}}$	$∆G_{131}^{\mathrm{AB}}$	$∆G_{131}^{\mathrm{EL}}$	$∆G_{131}$	$∆G_{132}^{\mathrm{LW}}$	$∆G_{132}^{\mathrm{AB}}$	$∆G_{132}^{\mathrm{EL}}$	$∆G_{132}$
**0**	−3.75	−1.63	0.12	−5.36	−4.23	−4.02	−0.03	−8.28
**2**	−4.25	9.22	0.08	5.05	−4.50	14.07	−0.01	9.96
**4**	−4.76	13.24	0.05	8.53	−4.76	11.07	0.02	8.23
**8**	−5.28	14.10	0.02	8.90	−5.01	13.89	0.03	8.91

**Table 2:** Interfacial condensation free energy and adhesion free energy of the ultrafiltration membrane surface, caused by simulated natural water (mJ/m^2^).

Dosage (mg/L)	$∆G_{131}^{\mathrm{LW}}$	$∆G_{131}^{\mathrm{AB}}$	$∆G_{131}^{\mathrm{EL}}$	$∆G_{131}$	$∆G_{132}^{\mathrm{LW}}$	$∆G_{132}^{\mathrm{AB}}$	$∆G_{132}^{\mathrm{EL}}$	$∆G_{132}$
**0**	−2.37	−6.03	0.11	−8.29	−3.36	1.30	−0.02	−2.04
**1**	−5.28	10.63	0.06	5.41	−5.01	14.85	0.02	9.89
**4**	−5.54	8.43	0.02	2.91	−5.14	13.19	0.03	8.11
**8**	−5.80	−7.35	−0.01	−13.16	−5.46	5.29	0.10	−0.07

## Data Availability

Some or all data, models, or code that support the findings of this study are available from the corresponding author upon reasonable request.
